# Reorienting in Virtual 3D Environments: Do Adult Humans Use Principal Axes, Medial Axes or Local Geometry?

**DOI:** 10.1371/journal.pone.0078985

**Published:** 2013-11-05

**Authors:** Althea H. Ambosta, James F. Reichert, Debbie M. Kelly

**Affiliations:** Department of Psychology, University of Manitoba, Winnipeg, Manitoba, Canada; University of Sussex, United Kingdom

## Abstract

Studies have shown that animals, including humans, use the geometric properties of environments to orient. It has been proposed that orientation is accomplished primarily by encoding the principal axes (i.e., global geometry) of an environment. However, recent research has shown that animals use local information such as wall length and corner angles as well as local shape parameters (i.e., medial axes) to orient. The goal of the current study was to determine whether adult humans reorient according to global geometry based on principal axes or whether reliance is on local geometry such as wall length and sense information or medial axes. Using a virtual environment task, participants were trained to select a response box located at one of two geometrically identical corners within a featureless rectangular-shaped environment. Participants were subsequently tested in a transformed L-shaped environment that allowed for a dissociation of strategies based on principal axes, medial axes and local geometry. Results showed that participants relied primarily on a medial axes strategy to reorient in the L-shaped test environment. Importantly, the search behaviour of participants could not be explained by a principal axes-based strategy.

## Introduction

Orientation is the initial phase of navigation whereby an individual determines a sense of heading or direction in which to begin travelling. When disoriented, two general types of visual cues that can be used to determine one's position are features and geometry. Featural cues are the distinct properties of objects or surfaces such as colour, pattern or texture, whereas geometric cues are the metric properties or relations between objects or surfaces such as distance, direction or angular information [1], [2]. Cheng [1] was the first to show that rats encoded environmental geometry to reorient despite the presence of salient featural cues. During a reference memory task, disoriented rats were trained to search for food that was consistently located in one corner of an enclosed rectangular environment. Visually distinct panels placed in each corner of the environment served as featural cues (along with distinctive olfactory information) and the differential lengths of the walls coupled with sense information served as geometric cues. After considerable training the rats eventually learned to search in the corner associated with the correct featural cue; however, they also committed rotational errors by searching in the corner diagonally opposite to the correct corner. This finding of systematic rotational errors supported that, despite the presence of seemingly more salient features, the rats showed a reliance on the geometric shape of the environment to reorient. Following Cheng's original investigation other studies have supported a widespread encoding of geometry by numerous other species (see [3] for a review).

**Figure 1:**
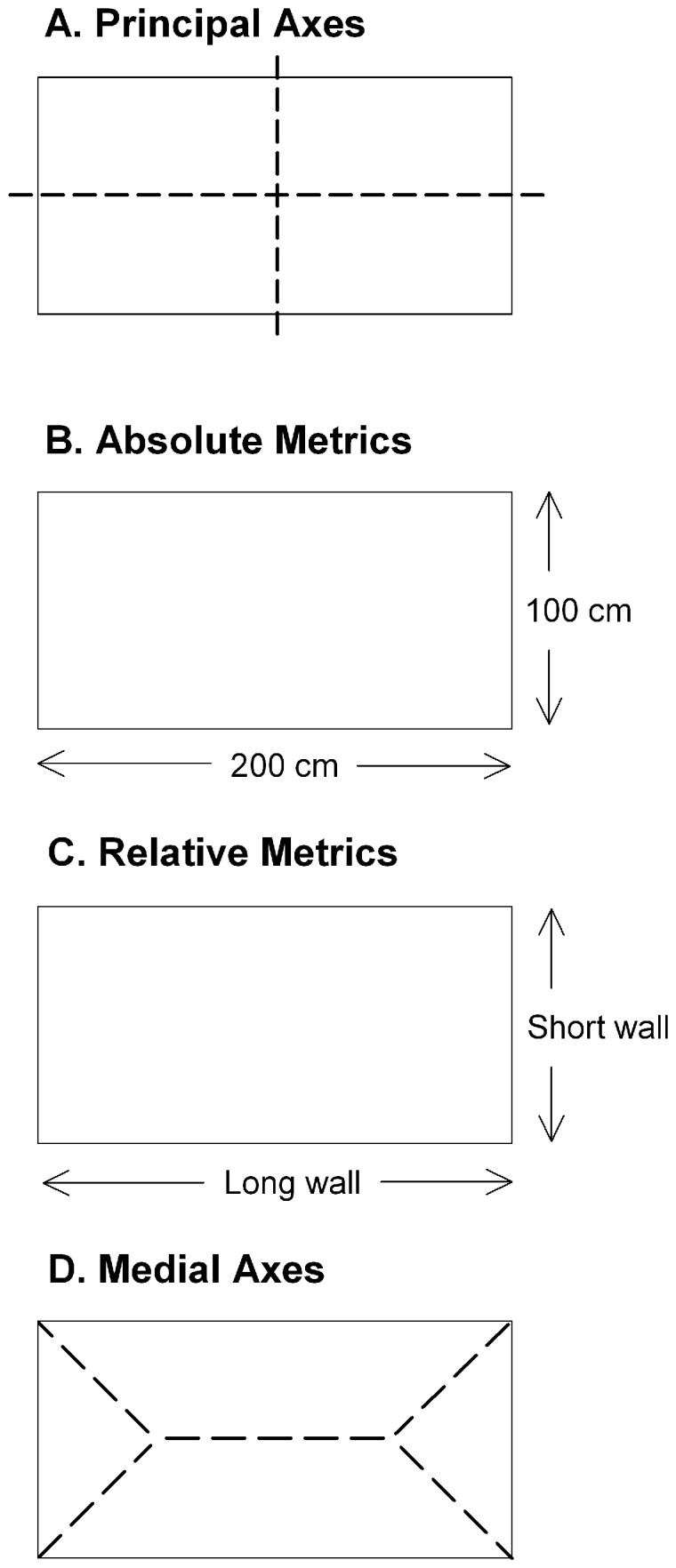
Schematics showing the geometric properties of a rectangular space. A) The major principal axis, based on the global shape of a space, passes lengthwise through the center of a space. The minor principal axis lies perpendicular to the major principal axis. Based on the principal axes alone, the shape of a space cannot be determined. B) Absolute metrics are the precise metrics of a given area. For instance, a rectangular space is composed of a set of opposite walls that are 200 cm in length and another set of opposite walls that are 100 cm in length. C) Relative metrics are the relational properties of a given area. For instance, a rectangular space is composed of a set of opposite walls that are longer and another set of opposite walls that are shorter in comparative length. D) The medial axes are based on local shape parameters; the trunk axis passes through the length of the space and the branch axes extend to the vertices of the space. Based on the medial axes alone, the shape of an area can be determined.

Since the pioneering work of Cheng [1], spatial reorientation via geometry has been thought to depend on the principal axes of an environment [4], [5]. The principal axes are defined mathematically as two perpendicular lines bisecting the centroid of a space, with the major principal axis extending through the length of the space such that “the perpendicular distances from points in the space to the axis are minimized, by the least-squares criterion” ([2], p. 8; see Figure 1A). It has been suggested that the systematic rotational errors made by rats supported an encoding of the environment's principal axes [4]. A search strategy based on principal axes and sense information (e.g., searching in a corner to the right side of the major principal axis) would have led the rats to search in the correct corner as well as the diagonally opposite corner.

Although Cheng's research introduced the possibility that global geometric cues based on principal axes can be used for reorientation, a second possibility is that local geometric properties of an environment can also be used by an animal to determine its position [6], [7]. Within enclosed environments the differential lengths of walls, in addition to the degree of angular amplitude of individual corners, can serve as informative local geometric cues. Moreover, these local geometric properties can be encoded in terms of either absolute or relative metrics [8], [9]. Encoding the absolute local properties of an environment would entail learning the precise metrics of a given area (Figure 1B) whereas encoding the relative properties of an environment would involve making relational comparisons between the metric properties of a given area (Figure 1C).

To examine the use of local geometry, Tommasi and Polli [7] used a novel transformational approach in which disoriented chicks were trained to find food in one corner of a featureless parallelogram-shaped enclosure that contained two types of local geometric cues: wall length and angular amplitude. The chicks were first trained to locate food hidden near either an acute- or an obtuse-angled corner of the environment. Upon learning the task, the chicks were subsequently tested in transformed environments that isolated the precise geometric properties that had been experienced during training. Results showed that the chicks could reorient according to either wall length or angular amplitude alone when tested in a rectangular- or rhombic-shaped environment, respectively. However, when tested in a mirror image parallelogram-shaped environment, a transformation that placed wall length in conflict with corner amplitude, both groups of chicks searched in the acute-angled corners. Chicks in the acute group searched using corner angle information whereas chicks in the obtuse group searched using wall length information. The authors concluded that the acute-angled corners may have been particularly salient local geometric cues for the chicks compared to the obtuse-angled corners. However, the search pattern of the chicks could also be explained by a reliance on principal axes cues in that the chicks always searched in corners of the test environments that preserved the same global position relative to the major principal axis as their training corner [5].

Pearce, Good, Jones and McGregor [6] used a transformational approach to examine whether the overall shape of an environment or local cues were relied upon by rats for reorientation. Using a Morris water maze, rats were trained to search for a hidden platform located in one corner of a featureless rectangular-shaped pool. Following training, the rats were tested in a kite-shaped pool to examine whether they had learned the global geometry (overall shape of rectangular pool) or the local geometry (wall lengths of correct corners along with sense information) of the training environment. A reliance on global geometry would result in a random pattern of searching by the rats since the overall shape of the rectangular training pool did not match that of the kite-shaped test pool, whereas a reliance on local geometry would result in the rats searching in one corner of the kite-shaped pool that matched the wall lengths and sense information of the correct training corner. Results showed that rats directed a majority of their searches to the geometrically correct corner as well as to the apex of the kite-shaped pool. The authors interpreted this behaviour as supporting a local geometric strategy, in that the rats searched for a long wall and subsequently turned left, thereby searching most frequently in the correct corner as well as the apex. Cheng and Gallistel [5] argued instead that these results may reflect a global geometric strategy as the rats could have searched for a corner that was to the far right of the principal axis, a strategy that would have led the rats to search at the geometrically correct corner as well as at the apex since no other corners were present in that region of space. Thus, although the search behaviour of the rats could have reflected a dependence on local geometry, their behaviour could also be explained more parsimoniously as a reliance on global geometry, encoded in relation to the principal axes.

In response to Cheng and Gallistel's [5] argument for a global geometric strategy, McGregor, Jones, Good and Pearce [10] employed an approach that more clearly dissociated the principal axes from local geometry during testing, such that searching according to global geometry would have led the animals to a different location within the environment compared to local geometry. Using a Morris water maze, rats were initially placed in a featureless pentagon-shaped pool and were trained to find a hidden escape platform in one corner that was enclosed by one long wall and one short wall. Following training the rats were tested in a rectangular-shaped pool, a transformation that caused a disassociation between the principal axes of the two environments. Results showed that the rats spent the greatest amount of time searching in the two corners that were correct according to local geometry as defined by wall length as opposed to the two corners that were correct according to global geometry as defined by the principal axes. Therefore, by clearly differentiating between global and local geometric strategies, McGregor et al. argued that the rats' search behaviour was dependent upon local geometry and could not be attributed to a principal axes-based strategy.

Recent evidence by Kelly, Chiandetti and Vallortigara [11] suggests that the medial axes of an environment may also be used to guide reorientation. Medial axes can be represented as a trunk and branch structure of axes; branch axes extend to the vertices of an environment from a central trunk axis and capture the local shape of a given area ([2]; see Figure 1D). Kelly et al. were the first to empirically examine whether birds could use the medial axes of an environment to reorient since the medial axes are thought to be “computationally cheap” ([2], p. 10) in comparison to local geometry and might therefore proffer a more parsimonious explanation for reorientation. Disoriented pigeons and chicks were trained to find food in two geometrically identical corners of a featureless rectangular-shaped environment. Following training, the birds were tested in an L-shaped environment, a transformation that dissociates principal axes, medial axes and local geometry-based strategies. Results showed that pigeons reoriented mainly according to medial axes cues whereas chicks relied primarily on local geometry (i.e., differential lengths of walls coupled with sense information) along with a secondary reliance on medial axes. Notably, neither the pigeons nor the chicks searched in a manner that was consistent with the use of a principal axes-based strategy. Although Cheng and Gallistel [5] initially proposed that animals may orient using medial axes, this research was the first to empirically show that medial axes could be used to explain reorientation behaviour.

Recent evidence using virtual environments suggests that adult humans may use principal axes to reorient [12]–[14]. However, none of these studies examined the possibility that participants may have been using medial axes cues. For example, Sturz et al. [13] trained adult humans to search in an obtuse-angled corner of a virtual trapezoid-shaped environment in which the correct corner could be identified by a nearby feature (colored orb), in addition to local geometry (i.e., obtuse-angled corner) and global geometry cues (i.e., position of goal corner relative to principal axes). When participants were tested in a featureless rectangular-shaped environment in which local geometric cues were no longer available (i.e., all corners were 90°), they searched in corners that maintained the same position relative to the principal axes as their training corner. Although the authors interpreted these findings as the use of principal axes, the possibility that participants may have been using medial axes was not specifically tested. In simple symmetric environments, such as the rectangular-shaped environment used during testing, a strategy based on either principal axes or medial axes would lead participants to search in the same location of the environment. However, in more complex asymmetric environments, the medial axes capture local shape information more precisely than do principal axes [2], [15].

**Figure 2:**
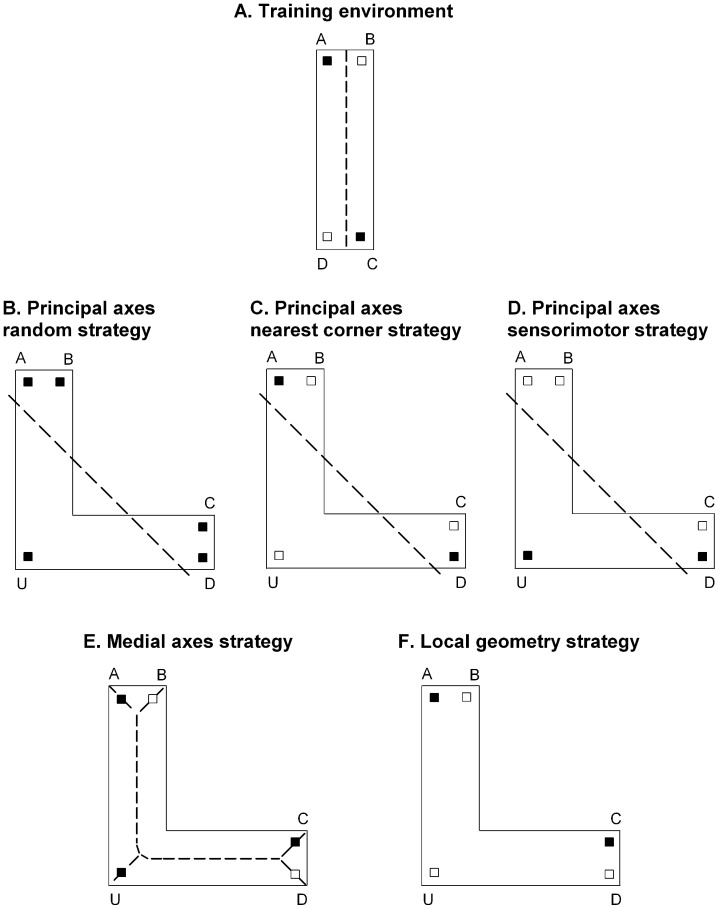
Predicted encoding strategies. As an example, each panel shows the corners that are predicted to be selected by group AC. A) A schematic representation of the training environment. The filled squares in the corners of the rectangle represent the response boxes that participants in group AC were trained to select. B–F) Schematic representations of the L-shaped environment. The filled squares in the corners of the L-shaped environment represent the response boxes that participants in group AC are predicted to select according to a given strategy. The open squares in the L-shaped environment represent the response boxes that participants should not select according to a given strategy. The dashed lines indicate the location of the major principal axis (B–D) and the medial axes (E).

**Figure 3:**
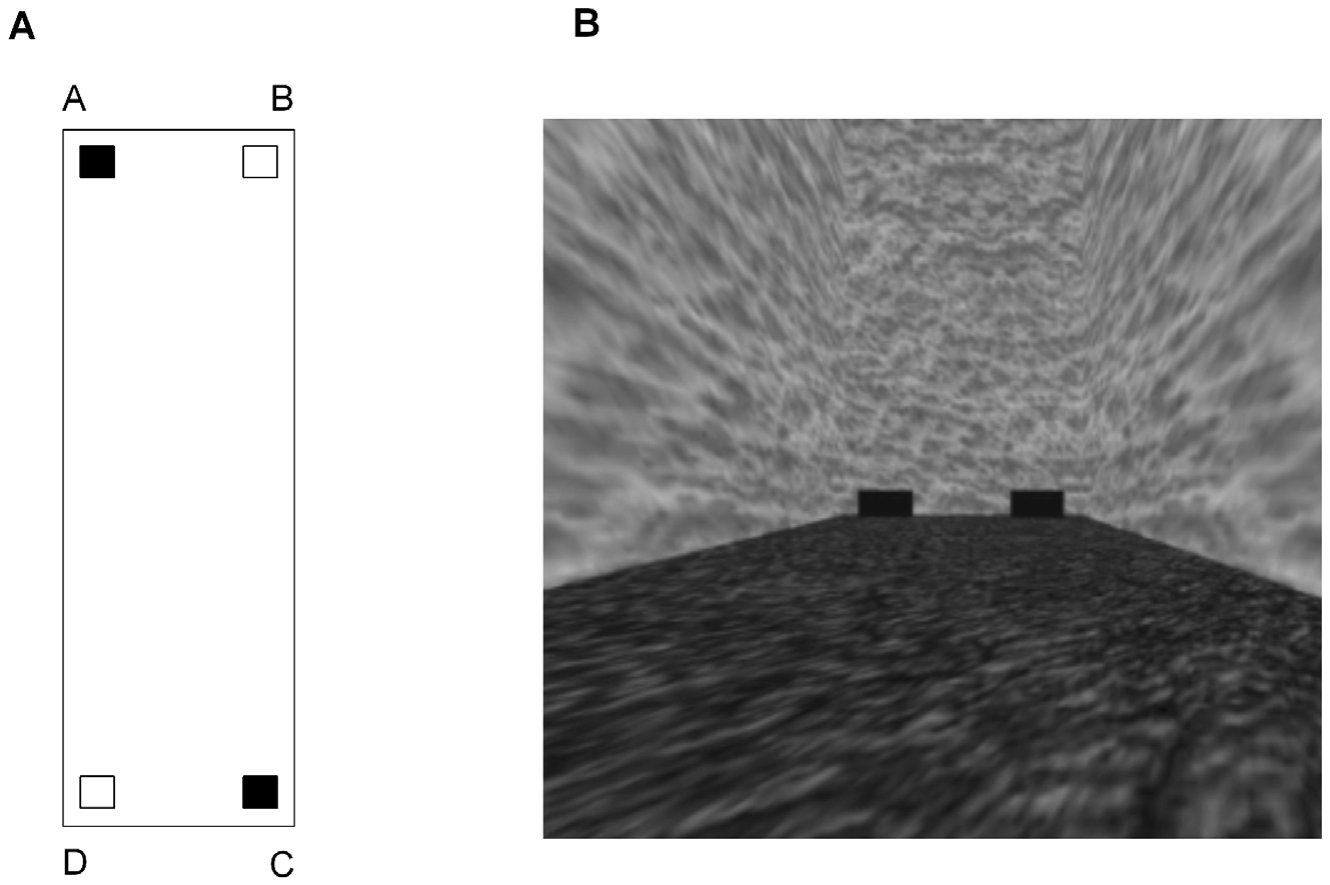
Training Environment. A) Schematic representation of the virtual training environment. Participants in Group AC were trained to choose either of the response boxes (indicated by the filled squares) located at the geometrically identical corners A and C whereas participants in Group BD were trained to choose either of the responses boxes (indicated by the open squares) located at corners B and D. B) One example viewpoint of the virtual training environment.

The current study examined the reorientation behaviour of adult humans using a virtual task that was similar in design to that employed by Kelly et al. [11]. Virtual environments devoid of informative featural cues were used during training and testing. Participants were trained to select response boxes located in geometrically identical corners of an enclosed rectangular environment (Figure 2A). Testing in an enclosed L-shaped environment allowed for a clear dissociation of search patterns based on principal axes, medial axes and local geometry, thereby providing a means for evaluating which strategy was used during reorientation. Since the global shape of the test environment did not match the training environment, we investigated three possible patterns of search behaviour which would indicate the use of principal axes: 1) random searching distributed equally among all corners because the principal axes does not directly coincide with any of the corners (principal axes random strategy; Figure 2B), 2) searching distributed equally between the two corners nearest to the major principal axis (principal axes nearest corner strategy; see Figure 2C), or 3) search guided by a sensorimotor pattern, such that the participant uses the principal axes as a guide to the nearest wall, at which point sense information is used to direct searching equally between the two corners at the ends of the principal axes that are correct according to sense information (principal axes sensorimotor strategy; see Figure 2D). We also investigated whether the pattern of search behaviour indicated the use of medial axes. In this case we would expect participants to distribute their search equally among the three corners indicated by the medial axes coupled with sense information (medial axes strategy; see Figure 2E). Finally, we investigated whether the pattern of search behaviour indicated the use of local geometric cues. In this case we would expect participants to distribute their search equally between the two corners that shared the same local geometric information as learned during training (local geometry strategy; see Figure 2F). The methods employed by this study allowed us to empirically evaluate whether men and women use principal axes, medial axes or local geometry when reorienting in space.

## Methods

### Participants

A total of 132 participants were recruited from the University of Manitoba's first-year undergraduate research participant pool in exchange for course credit. Data was analyzed only for those participants who successfully passed training and completed the experiment (n = 120; 60 men, 60 women; Mean age = 19.5 years). Participants were randomly assigned to one of two groups (group AC or group BD) based upon the location of geometrically identical corners that they were trained to select.

### Ethics Statement

Informed written and verbal consent was provided by all participants prior to beginning the experiment. All procedures and protocol were approved by the University of Manitoba's Psychology Research Ethics Board.

### Apparatus

Virtual training and testing environments were created using Microsoft Visual Studio Express 2010 and were presented to participants using a desktop computer (Processor speed: 2.66 GHz, Graphics Card: NVIDIA GeForce 9500 GT) with a 17 inch LCD monitor (1440×900 pixels). Each participant navigated the virtual environment via a first-person perspective using a keyboard and mouse. Headphones worn by participants provided auditory feedback during trials.

### Virtual Environments

#### Training Environment

Training took place in a rectangular-shaped virtual environment (200 virtual units (vu) length x 50 vu width). The height of the camera was maintained at a viewing perspective that was the average height of a human (1.6 meters) or 16 vu from the floor of the room; the virtual room in real world units would be approximately 20 meters×5 meters, length x width, respectively. The walls of the training environment were uniform gray and the floor was dark green. Located equidistant from each corner was an identical blue response box (10 length×10 width×7 height vu) which participants could select to register their response (see Figure 3A for a schematic representation of the environment and Figure 3B for an example of the environment from the participant's viewpoint).

#### Testing Environment

**Figure 4:**
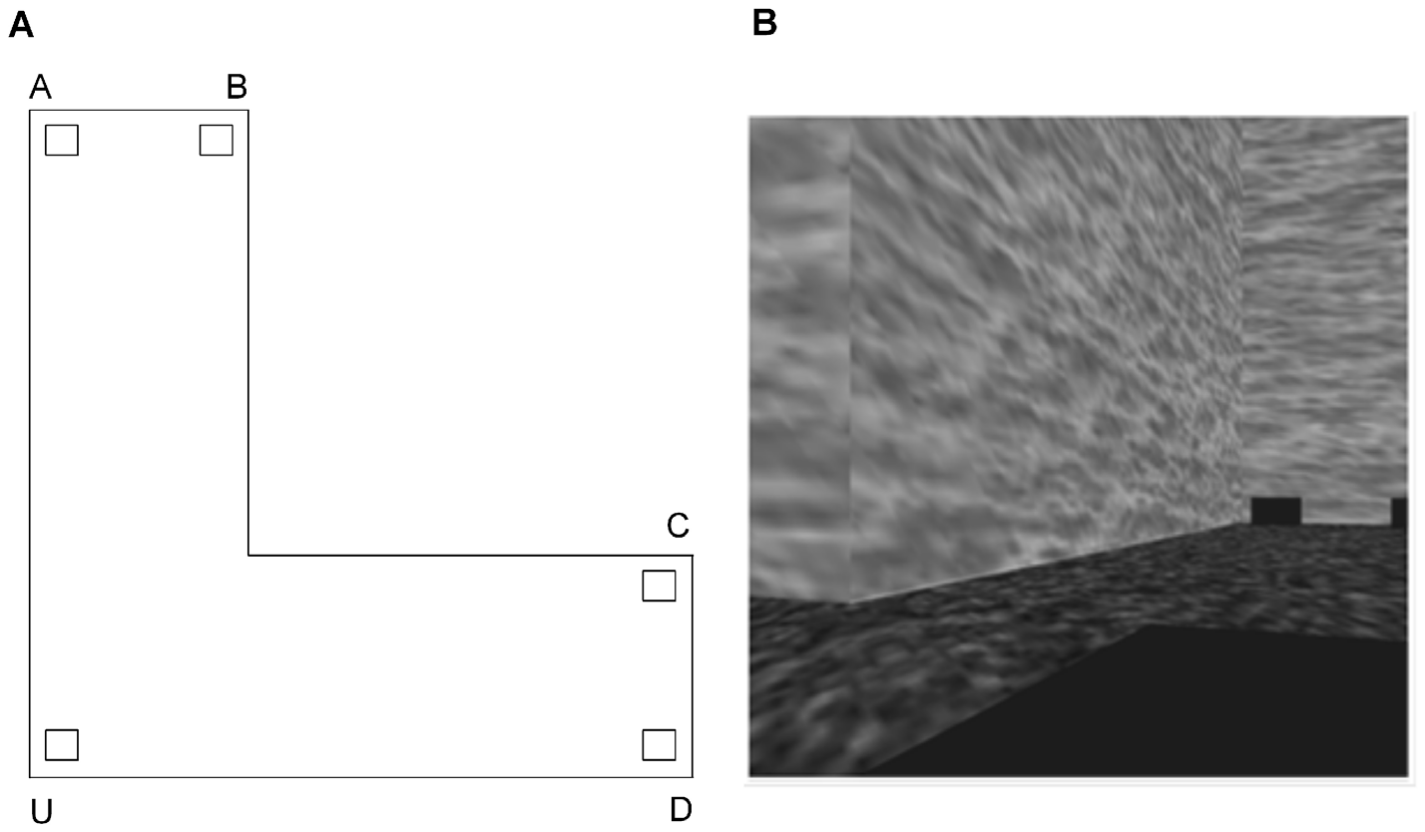
Testing Environment. A) Schematic representation of the virtual L-shaped testing environment. The open squares represent the response boxes located at each corner. B) One example viewpoint of the virtual testing environment shown from corner U.

Testing was conducted in an L-shaped virtual environment in which the two longest walls were 200 vu, the two intermediate walls were 150 vu and the two shortest walls were 50 vu. The wall and floor colours of the testing environment were identical to that of the training environment. Response boxes identical to those in the training environment were placed equidistant from the five vertices of the L-shaped environment (see Figure 4A for a schematic representation of the environment and Figure 4B for an example of the environment from the participant's viewpoint).

### Training Procedures

At the beginning of each trial, the participant was positioned in the middle of one of the four walls within the environment; each wall was used as a starting position an equal number of times. During training participants learned to move through the virtual environment by using the arrow keys on a keyboard and a mouse to manipulate the viewpoint. Participants learned to approach different corners and choose the response boxes situated in the corners. In order to choose a response box in a particular corner the participant approached that corner and hovered over the corresponding response box until the response box changed color from blue to white. Once white, the participant pressed the spacebar on the keyboard to select that particular response box. Headphones worn by participants provided auditory feedback regarding the correctness of the choice; a high-pitched bell sound signalled a correct choice (“correct sound”) and a low-pitched thud sound signalled an incorrect choice (“incorrect sound”). There were always two correct response boxes within the rectangular training room, both of which were located in geometrically identical corners (corners A and C for group AC and corners B and D for group BD; see Figure 3A, the filled boxes and open boxes for the two groups, respectively). If a correct response box was selected, the correct sound was played through the headphones and the trial ended. Once a trial ended, participants were placed in a different location in the training environment and were again required to find a correct response box. If an incorrect response box was selected, the incorrect sound was played thorough the headphones and the participant was given additional opportunities to find a correct response box. Participants were not given a time limit during each trial, as training trials could only be completed through the selection of a correct response box.

Although training was continuous, for the purpose of calculating choice accuracy, trials were grouped in blocks consisting of eight trials per block (each of the four starting positions were repeated twice per block). Participants who chose a correct response box as their first choice during seven of the eight trials within a training block advanced to testing; participants who did not achieve this criterion experienced an additional training block which was comprised of eight trials and accuracy was recalculated based on the last completed block of trials. Participants continued to experience additional training blocks until they either successfully advanced to testing or until 30 minutes had elapsed. Those participants who did not successfully advance to testing were thanked for their participation but their data were not used for analyses.

### Testing Procedures

Participants who successfully met the training criterion were directly transitioned to the testing phase of the experiment. During the testing phase, participants experienced three types of trials: *training, control*, and *test*, with the order of presentation pseudo-randomized such that test and/or control trials were not presented consecutively. During this phase, 24 training trials (4 starting positions x 6 repetitions) were presented for the purpose of providing continued auditory feedback to prevent extinction. There were a total of 12 control trials (4 starting positions×3 repetitions) that were identical to training trials except that auditory feedback was not provided following a choice (i.e., choices were not reinforced) and the trial terminated after the participant made a single choice. There were a total of 12 test trials that occurred in the L-shaped environment (6 starting positions, one position from the middle of each wall in the environment x 2 repetitions). Auditory feedback was not provided following a choice (i.e., choices were not reinforced) and the trial terminated after the participant made a single choice.

### Predicted Reorientation Strategies

We examined five possible reorientation strategies: a *principal axes strategy* (used in one of three possible ways: *random, nearest corner* and *sensorimotor*), a *medial axes strategy* and a *local geometry strategy*. If participants were relying on the principal axes of the test environment to reorient, their search behaviour would result in one of three possible patterns. First, neither of the principal axes leads directly to a goal location in the testing environment. Therefore, if the participants were relying on the principal axes we would expect them to search randomly among the five response boxes, herein referred to as the “principal axes random strategy” (see Figure 2B). Second, the participants may use the major principal axis to direct them to the corners nearest to the location where the major principal axis intersects with a wall. The use of this strategy should lead participants in both groups to select only the response boxes located in corners A and D, herein referred to as the “principal axes nearest corner strategy” (see Figure 2C). Third, the participants may use the major principal axis along with the sensorimotor response learned during training (i.e., turn left or turn right). The use of this strategy should lead participants in group AC to choose equally between the response boxes located at corners U and D (see Figure 2D) whereas participants in group BD should choose equally between the response boxes located at corners U and A, herein referred to as a “principal axes sensorimotor strategy”.

If the participants were relying on the medial axes of the test environment to reorient, their search behaviour should be guided by the branches of the medial axes coupled with correct sense information (i.e., left or right) as learned during training. The use of this strategy should lead participants in group AC to select equally among the response boxes located in corners A, C and U (see Figure 2E), whereas participants in group BD should select equally among the response boxes located in corners B, D and U, herein referred to as a “medial axes strategy”.

Finally, if the participants were relying on the local geometric cues, their search behaviour should be guided by the wall lengths coupled with correct sense information as learned during training. The use of this strategy should lead participants in group AC to select equally between the response boxes located in corners A and C, whereas participants in group BD should select equally between the response boxes located in corners B and D, as these are the only two corners which are subtended by walls with the correct relative metrics (see Figure 2F). This strategy is herein referred to as the “local geometry strategy”.

## Results

Analyses are based on data collected from the 120 participants (60 men, 60 women) who completed testing. For training and control data, group and gender differences were analyzed using an ANOVA. For testing data, an ANOVA was used to examine choice accuracy with corner choice (corners A, B, C, D or U) and gender (men or women) as variables. Separate ANOVAs were conducted for groups AC and BD since the corners that were predicted to be chosen according to a specific strategy differed between the two groups. Main effects and interactions were analyzed using Tukey-Kramer tests. To compensate for the number of analyses conducted, the alpha level for all tests was set to p<.01.

### Training Trials

In order to examine differences in the number of training blocks needed to reach criterion, a two-way between subjects ANOVA was used to analyze group (AC and BD) and gender (male and female) differences. There was no effect of group, F(1, 116) = .010, p = .910, showing no difference between groups AC (M = 2.083, SEM = .209) and BD (M = 2.117, SEM = .209) in the number of training blocks required to reach criterion. Similarly, there was no effect of gender, F(1, 116) = .460, p = 501, showing that there were no differences between the number of training blocks needed by men (M = 2.000, SEM = .210) and women (M = 2.200, SEM = .210) to learn the task. The interaction between group and gender was not significant F(1, 116) = .010, p = .911.

### Control Trials

In order to analyze group and gender differences in the proportion of geometrically correct choices made during non-reinforced control trials, a two-way between subjects ANOVA was used. There was no effect of group, F(1, 116) = 4.250, p = 0.042, showing no difference between groups AC (M = .950, SEM = .014) and BD (M = 0.907, SEM = .014) in the proportion of geometrically correct choices made during control trials. Furthermore, there was no effect of gender, F(1, 116) = .990, p = .321, showing no difference between men (M = .938, SEM = .014) and women (M = .918, SEM = .014) in the proportion of geometrically correct choices made during control trials. The interaction between group and gender was also not significant, F (1, 116) = 4.250, p = .041. Overall, men and women, regardless of group assignment distributed over 90% of their choices to the geometrically correct corners during non-reinforced control trials.

### Test Trials

**Figure 5:**
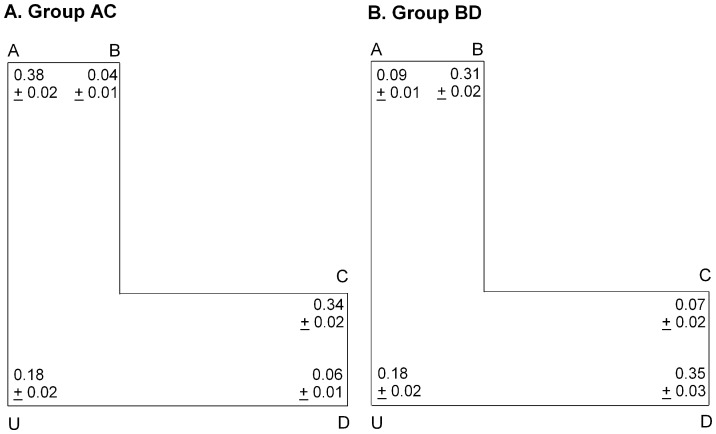
Performance during testing in the L-shaped arena. The proportion of choices (mean and standard error of the mean) made to each of the corners in the L-shaped environment across all testing trials.

Data for all analyses are shown in Figure 5A for group AC and Figure 5B for group BD. Choice accuracy during testing was examined for groups AC and BD using separate ANOVAs with corner choice (corners A, B, C, D or U) as the within-subject variable and gender (men or women) as the between-subject variable. Results for group AC showed a significant main effect of corner choice, F(4, 290) = 94.110, p<.001, but no effect of gender, F(1, 290) = .000, p = 1.000 and no interaction between corner choice and gender, F(4, 290) = 1.440, p = .222. Similarly, results for group BD showed a significant main effect of corner choice, F(4, 290) = 41.610, p<.001, but no effect of gender, F(1, 290) = .000, p = 1.000 and no interaction between corner choice and gender, F(4, 290) = .660, p = .617.

#### Principal axes random strategy

To determine whether participants were relying on a principal axes random strategy, we examined whether choices were equally distributed among the five corners of the L-shaped test environment. Results indicated that there was a main effect of corner choice for both groups AC and BD showing that the groups did not distribute their choices equally among the five corners of the test environment (group AC: F(4, 290) = 94.110, p<.001; group BD: F(4, 290) = 41.610, p<.001; see Figure 5). Thus, the use of a principal axes random strategy was not supported.

#### Principal axes nearest corner strategy

To determine whether participants were relying on a principal axes nearest corner strategy, a one-sample t-test was used to analyze whether the sum of choices to the two corners A and D were above the level of chance (chance = .40) for each group. Results showed that the sum of choices to corners A and D did not differ from chance for participants in group AC (M = .439, SEM = .016), t(59) = 2.300, p = .025 or group BD (M = .443, SEM = .023), t(59) = 1.867, p = .067. Thus, these results do not support the use of a principal axes nearest corner strategy for reorientation.

#### Principal axes sensorimotor strategy

To determine whether participants were relying on a principal axes sensorimotor strategy, a one-sample t-test was used to analyze whether the sum of choices to the two predicted corners were above the level of chance (chance = .40) for each group. Results showed that participants in group AC selected corners U and D (M = .246, SEM = .024) significantly below the level of chance, t(59) = −6.432, p<.001. Participants in group BD were also found to select the predicted corners A and U (M = .268, SEM = .026) significantly below the level of chance, t(59) = −5.006, p<.001. Thus, these results do not support the use of a principal axes sensorimotor strategy for reorientation.

#### Medial axes strategy

To determine whether participants were relying on a medial axes strategy, a one-sample t-test was used to analyze whether the sum of choices to the three predicted corners were above the level of chance (chance = .60) for each group. Results showed that participants in group AC selected the predicted corners A, C and U (M = .901, SEM = .017) significantly above chance, t(59) = 18.253, p<.001. Similarly, participants in group BD selected the predicted corners B, D and U (M = .840, SEM = .024) significantly above the level of chance, t(59) = 9.962, p<.001. To support the use of a medial axes strategy, whereby the predicted corners each have equal excitatory properties, the proportion of the choices should be equally distributed among the three corners. Results of Tukey-Kramer tests showed that participants in groups AC and BD did not direct an equal proportion of choices to the three predicted corners (group AC: choices to corner A (M = .376, SEM = .020) and corner C (M = .342, SEM = .020) differed significantly from corner U (M = .183, SEM = .020), ps<.010; group BD: choices to corner B (M = .310, SEM = .020) and corner D (M = .353, SEM = .030) differed significantly from corner U (M = .178, SEM = .020), ps<.010).

Further, we examined whether the proportion of choices to corner U differed from those to the two incorrect corners (i.e., corners B and D for group AC and corners A and C for group BD). We hypothesize that if the participants were using a medial axes strategy, responses to corner U should be greater than to either of the corners that were incorrect. Results of Tukey-Kramer tests showed that participants in group AC selected corner U (M = .183, SEM = .020) significantly more than the incorrect corners B (M = .036, SEM = .010), and D (M = .062, SEM = .010), p's<.010. Participants in group BD selected corner U (M = .178, SEM = .020) significantly more than the incorrect corner C (M = .069, SEM = .020), p<.010 but not A (M = .090, SEM = .010), p>.010. Therefore, these results partially support the use of a medial axes strategy.

#### Local geometry strategy

To determine whether participants in each group were relying on local geometry to reorient, Tukey-Kramer tests were used to compare the proportion of choices made to each of the corners that were correct according to local geometry (corners A and C for group AC and corners B and D for group BD) with the proportion of choices to corner U, a corner that was partially correct since it was subtended by one wall with correct length but the other wall with an incorrect length. Participants in group AC were found to select corner A as well as corner C significantly more than corner U (group AC: choices to corner A (M = .376, SEM = .020) and corner C (M = .342, SEM = .020) differed significantly from corner U (M = .183, SEMU = .020), ps<.010). Participants in group BD were found to select corner B as well as corner D significantly more than corner U (group BD: choices to corner B (M = .310, SEM = .020) and corner D (M = .353, SEM = .030) differed significantly from corner U (M = .178, SEM = .020), ps<.010). Thus our results support the use of a local geometry strategy for reorientation.

Next, to determine whether there were differences in the proportion of choices to the two predicted corners, Tukey-Kramer tests were conducted for each group. Participants in both groups were found to direct a similar proportion of choices to the predicted corners (group AC: A (M = .376, SEM = .020) versus C (M = .342, SEM = .020), p = .110; group BD: B (M = .310, SEM = .020) versus D (M = .353, SEM = .030), p = .190). Therefore, these results suggest the use of a local cues strategy.

We might conclude that the participants were using a combined strategy of local cues and medial axes. However, another possibility is that the participants were only relying on medial axes but had learned to approach the corners associated with reward and inhibit the corners not associated with reward. Using group AC as an example, excitation would cause the participants to select corners A and C during the majority of trials (this is supported by our analyses of a local cues strategy). Whereas, corner U, however, would have both excitatory and inhibitory properties; choices to this corner would be intermediate (this is supported by our analyses of a medial axes strategy). Therefore, to evaluate which of these strategies (local cues and medial axes versus medial axes with excitatory and inhibitory properties) best predicts our data, we compared the two strategies using the Akaike information criterion (AIC) [16]. The AIC showed that the medial axes strategy driven by excitation and inhibition provided a better fit than a combined local geometry and medial axes strategy (Table 1). Furthermore, a medial axes strategy would offer a more parsimonious explanation of our results.

**Table 1:** Model comparisons.

Model	r	*M*error^2^	AIC
Local Geometry and Medial Axes	2	.0208	−193.929
Medial Axes (Excitation and Inhibition)	1	.0929	−117.839

The Akaike Information Criterion (AIC) provides a means for ranking models based on the number of parameters used to fit the data (r) and the residual error (*M*error^2^), [26]. AIC = nlog(error) +2(r +2), where n is the number of data points and r is the number of free parameters. Error = *M*error^2^(n−r−1)/n.

## Discussion

The current study was the first to examine whether adult humans encode the geometry of an environment using principal axes, medial axes or local geometry. Employing a design similar to that of Kelly et al. [11], participants were first trained to select geometrically equivalent corners in a rectangular-shaped virtual environment, and were subsequently tested in an L-shaped virtual environment in order to dissociate reorientation strategies based on principal axes, medial axes and local geometry. Our results showed that participants did not reorient according to a principal axes strategy but instead relied on a medial axes strategy. Our findings did not show differences between men and women in the ability to encode the geometry of the training environment or in their reliance on medial axes information.

By using an L-shaped environment during testing we were able to account for all possible principal-axes based strategies that participants may have used, and in no case did we find support for the use of principal axes. Although recent studies have suggested that humans may reorient in virtual environments according to principal axes cues [12]–[14], [17], these studies did not thoroughly distinguish between principal axes and medial axes-based strategies. Therefore, the possibility remains that participants in these studies may have been influenced by medial axes instead of principal axes. Why did the participants in our study not use principal axes for reorientation? Indeed, the prime advantage of such a strategy is that it would allow an individual to parsimoniously encode the entirety of a space using minimal cognitive resources. However, as Cheng [2] observed, principal axes on their own are not ideal descriptors of the shape of a space. This shortcoming is further accentuated in more complex environments, thus making the encoding of principal axes, at the very least, an unreliable strategy across a diverse range of environments [15]. Organisms may forego the cognitive simplicity inherent to principal axes encoding and instead rely on a more informative locally-based strategy.

Research examining navigation by insects has shown that terrestrial landmarks as well as the surrounding visual panorama are used for relocating a goal position [18], [19], [20]. Although it is clear that insects make use of landmarks, there is debate as to whether the visual scene provides a type of context in which landmark information is extracted, or whether the panoramic scene itself including landmarks are integrated or “bound” [21], [22], [23]. Much of this research has focused on navigation by ants, with near panoramic visual abilities; therefore, it will be important to examine these issues across different taxa [24], [25]. Although humans are clearly capable of encoding complex allocentric spatial relations between objects and surfaces, the degree to which independently stored viewpoints facilitate this process is not fully understood. Understanding how a range of animals encode information from their environments will allow for the examination of the underlying mechanisms involved in spatial navigation [11], [15], [22], [23], [26], [27].

A growing number of studies have shown that reorientation by human and non-human animals is highly sensitive to alterations in local geometry and our results add to this body of research [7], [10], [11], [28], [29]. During testing in the L-shaped environment, participants directed the majority of their searches to the corners that matched the medial axes of the training environment. Medial axes are defined locally, thus the orientation and position of these axes do not change when the definition of the global space is altered [15]. In contrast, the position and orientation of principal axes can change based upon the shape of an environment. Thus, it is possible that medial axes are relied upon as a stable referencing system that provides sufficient information about the shape of a space [2], [15], unlike principal axes, but is more stable than reliance on local geometric cues alone. Furthermore, medial axes have been suggested to be computationally economical [2].

Although the results of our study are in agreement with those of pigeons by Kelly et al. [11] for which the primary reorientation strategy was based on medial axes, they may conflict with the findings of chicks for which the primary reorientation strategy was based on a combined strategy of local geometric cues and medial axes. Why differences (and similarities) might exist in the use of medial axes cues across species will clearly benefit from further comparative study.

Since gender differences have previously been observed during navigational tasks [30], reorientation tasks [31], and perhaps most robustly with mental rotation tasks [32], [33], we were curious as to whether men and women would show a differential reliance on local or global geometry for reorientation. Our results did not show gender differences; both men and women relied equally on medial axes cues for reorientation. As well, neither men nor women showed a reliance on any of the principal axes-based strategies to reorient. Although incidental geometric encoding by women and men differs when participants are trained with both geometry and distinctive features present [31], these gender differences are absent when men and women are trained exclusively with environmental geometry [28]. Our results support these findings and show that men and women encode medial axes information with equal facility when geometry is the only cue made available during training.

Contrary to the long-held assumption that animals reorient according to the principal axes of their environment, our study showed for the first time that humans rely primarily on medial axes information for reorientation in a virtual environment. The use of medial axes cues by the different species tested thus far, suggests a quantitative rather than a qualitative difference in the geometric strategies used for reorientation.

## References

[1] Cheng K (1986) A purely geometric module in the rat's spatial representation. Cognition 23: 149–178. 3742991 10.1016/0010-0277(86)90041-7

[2] Cheng K (2005) Reflections on geometry and navigation. Connect Sci 17: 5–21.

[3] Cheng K, Newcombe NS (2005) Is there a geometric module for spatial orientation? Squaring theory and evidence. Psychon B Rev 12: 1–23. 10.3758/bf03196346 15945200

[4] Gallistel CR (1990) The Organization of Learning. Cambridge, MA: Bradford Books/MIT Press.

[5] Cheng K, Gallistel CR (2005) Shape parameters explain data from spatial transformations: comment on Pearce et al. (2004) and Tommasi & Polli (2004). J Exp Psychol Anim B 31: 254–259. 10.1037/0097-7403.31.2.254 15839781

[6] Pearce JM, Good MA, Jones PM, McGregor A (2004) Transfer of spatial behavior between different environments: implications for theories of spatial learning and for the role of the hippocampus in spatial learning. J Exp Psychol Anim B 30: 135–147. 10.1037/0097-7403.30.2.135 15078123

[7] Tommasi L, Polli C (2004) Representation of two geometric features of the environment in the domestic chick (*Gallus gallus*). Anim Cogn 7: 53–59. 12884079 10.1007/s10071-003-0182-y

[8] Kelly DM, Spetch ML (2001) Pigeons encode relative geometry. J Exp Psychol Anim B 27: 417–422. 11676090

[9] Spetch ML, Cheng K, MacDonald SE, Linkenhoker BA, Kelly DM, et al (1997) Use of a landmark configuration in pigeons and humans: II. Generality across search tasks. J Comp Psychol 111: 14–24.

[10] McGregor A, Jones PM, Good MA, Pearce JM (2006) Further evidence that rats rely on local rather than global spatial information to locate a hidden goal: reply to Cheng and Gallistel (2005). J Exp Psychol Anim B 32: 314–321. 10.1037/0097-7403.32.3.314 16834498

[11] Kelly DM, Chiandetti C, Vallortigara G (2011) Re-orienting in space: do animals use global or local geometry strategies? Biol Letters 7: 372–375. 10.1098/rsbl.2010.1024 PMC3097861 21159689

[12] Bodily KD, Eastman CK, Sturz BR (2011) Neither by global nor local cues alone: evidence for a unified orientation process. Anim Cogn 14: 655–674. 21509592 10.1007/s10071-011-0401-x

[13] Sturz BR, Forloines MR, Bodily KD (2012) Enclosure size and the use of local and global geometric cues for reorientation. Psychon B Rev 19: 270–276. 10.3758/s13423-011-0195-5 22218783

[14] Sturz BR, Gurley T, Bodily KD (2011) Orientation in trapezoid-shaped enclosures: implications for theoretical accounts of geometry learning. J Exp Psychol Anim B 37: 246–253. 10.1037/a0021215 21319918

[15] Kelly DM, Durocher S (2011) Comparing geometric models for orientation: medial vs. principal axes. Commun Integr Biol 4: 710–712. 22446534 10.4161/cib.17318 PMC3306338

[16] Burnham KP, Anderson DR (2002) Model selection and multi- model inference: a practical information-theoretic approach (2nd ed.). Springer-Verlag.

[17] Sturz BR, Bodily KD (2011) Is surface-based orientation influenced by a proportional relationship of shape parameters? Psychon B Rev 18: 848–854. 10.3758/s13423-011-0111-z 21604095

[18] Sturzl W, Cheung A, Cheng K, Ziel J (2008) The information content of panoramic images I: the rotational errors and the similarity of views in rectangular experimental arenas. J Exp Psychol Anim B 34: 1–14. 10.1037/0097-7403.34.1.1 18248111

[19] Cheung A, Sturzl W, Zeil J, Cheng K (2008) The information content of panoramic images II: view-based navigation in nonrectangular experimental arenas. J Exp Psychol Anim B 34: 15–30. 10.1037/0097-7403.34.1.15 18248112

[20] Graham TS, Cheng K (2009) Ants use the panoramic skyline as a visual cue during navigation. Curr Biol 19: R935–R937. 19889365 10.1016/j.cub.2009.08.015

[21] Wystrach A, Beugnon G, Cheng K (2011) Landmarks or panoramas: what do navigating ants attend to for guidance? Front Zool 8: 21. 21871114 10.1186/1742-9994-8-21 PMC3177867

[22] Zeil J (2012) Visual homing: an insect perspective. Curr Opin Neurobiol 22: 285–293. 22221863 10.1016/j.conb.2011.12.008

[23] Lent DD, Graham P, Collett TS (2013) Visual scene perception in navigating wood ants. Curr Biol 23: 684–690. 23583550 10.1016/j.cub.2013.03.016

[24] Pecchia T, Gagliardo A, Vallortigara G (2011) Stable panoramic views facilitate snap-shot like memories for spatial reorientation in homing pigeons. PLoS ONE 6: e22657. 21818360 10.1371/journal.pone.0022657 PMC3144919

[25] Pecchia T, Vallortigara G (2010) View-based strategy for reorientation by geometry. J Exp Biol 213: 2987–2996. 20709927 10.1242/jeb.043315

[26] Epstein RA (2008) Parahippocampal and retrosplenial contributions to human spatial navigation. Trends Cogn Sci 12: 388–396. 18760955 10.1016/j.tics.2008.07.004 PMC2858632

[27] Wystrach A, Graham P (2012) What can we learn from studies of insect navigation? Anim Behav 84: 13–20.

[28] Lubyk DM, Dupuis B, Gutiérrez L, Spetch ML (2012) Geometric orientation by humans: angles weigh in. Psychon B Rev 19: 436–442. 10.3758/s13423-012-0232-z 22382695

[29] Lubyk DM, Spetch ML (2012) Finding the best angle: pigeons (*Columba livia*) weight angular information more heavily than relative wall length in an open-field geometry task. Anim Cogn 15: 305–312. 21918871 10.1007/s10071-011-0454-x

[30] Sandstrom NJ, Kaufman J, Huettel S (1998) Males and females use different distal cues in a virtual environment navigation task. Cognitive Brain Res 6: 351–360. 10.1016/s0926-6410(98)00002-0 9593991

[31] Kelly DM, Bischof WF (2005) Reorienting in images of a three-dimensional environment. J Exp Psychol – Hum L 31: 1391–1403. 10.1037/0096-1523.31.6.1391 16366797

[32] Parsons TD, Larson P, Kratz K, Thiebaux M, Bluestein B, et al (2004) Sex differences in mental rotation and spatial rotation in a virtual environment. Neuropsychologia 42: 555–562. 14728927 10.1016/j.neuropsychologia.2003.08.014

[33] Vandenberg SG, Kuse AR (1978) Mental rotations, a group test of three-dimensional spatial visualization. Percept Motor Skill 47: 599–604. 10.2466/pms.1978.47.2.599 724398

